# Impact of New Technologies for Middle-Aged and Older Patients: In-Depth Interviews With Type 2 Diabetes Patients Using Continuous Glucose Monitoring

**DOI:** 10.2196/10992

**Published:** 2019-02-21

**Authors:** Ching-Ju Chiu, Yu-Hsuan Chou, Yen-Ju Chen, Ye-Fong Du

**Affiliations:** 1 Institute of Gerontology College of Medicine National Cheng Kung University Tainan Taiwan; 2 Department of Medicine College of Medicine National Cheng Kung University Tainan Taiwan; 3 Division of Endocrinology and Metabolism Department of Internal Medicine National Cheng Kung University Hospital Tainan Taiwan

**Keywords:** diabetes mellitus, type 2, blood glucose, middle aged, aged, biomedical technology, Taiwan, qualitative research

## Abstract

**Background:**

Continuous glucose monitoring (CGM) uses subcutaneous sensors and records the average interstitial sensor current every 5 min in the recorder; data are subsequently exported to a computer 4 to 7 days later when calibration with self-measured blood glucose is made retrospectively. How middle-aged and older patients perceive the added technology intervention is not clear.

**Objective:**

The study aimed to understand the factors associated with the adoption of new technology in diabetes care, to understand the feelings and behaviors while using it, and to determine the changes in attitudes and behavior after completing the use of the new technology at the 3-month follow-up.

**Methods:**

Middle-aged and older type 2 diabetes patients who had received professional continuous glucose monitoring (iPro 2 [Medtronic]) were invited for semistructured in-depth interviews on the day of the CGM sensor removal and at 3 months after CGM-based counseling. A phenomenography approach was used to analyze the interview data.

**Results:**

A total of 20 type 2 diabetes patients (aged 53 to 72 years, 13 males and 7 females, 4 to 40 years duration of diabetes, mean glycated hemoglobin 8.54% [SD 0.71%]) completed 2 sections of semistructured in-depth interviews. Physician guidance and participant motivation toward problem solving were found to be factors associated with adoption of the device. Participants indicated that technology can be a reminder, a supervisor, and a visualizer of blood glucose, all of which are helpful for disease management. However, CGM is somewhat inconvenient, and some participants also reported that the provision of this new technology might be a hint of disease progression. There was a higher percentage of women compared with men who reported that CGM can be a reminder or a supervisor to help them with diet control.

**Conclusions:**

Physician guidance and participants’ degree of motivation are keys to adopting new technology in the case of middle-aged and older adults. Although the CGM sensor may cause inconvenience to patients on their limited body movement when wearing the device, it is helpful for diet control and is an effective behavioral modification tool that offers support, especially in the case of women.

## Introduction

### Background

Type 2 diabetes is a highly prevalent disease that increases in frequency with age. In Taiwan, diabetes ranks fifth [1] among the top 10 leading causes of death, and the prevalence of diagnosed type 2 diabetes is over 20% of the population over the age of 60 years [2]. In the future, the proportion of type 2 diabetes will continue to rise as the elderly population increases. In Taiwan, care and management practices for type 2 diabetes are basically consistent with the standards set by the American Diabetes Association, which emphasizes the importance of blood glucose management and blood glucose monitoring [3]. Maintaining normal blood glucose levels can prevent damage to the retina, kidneys, and other organs [4]. It has also been suggested in the past that a 1% reduction in glycated hemoglobin (HbA_1c_) can reduce other complications by 14% to 37% [5].

Control of blood glucose is assisted through self-monitoring of blood glucose levels. Generally, blood glucose measurement with a glucose meter allows patients to determine their current blood glucose levels. However, even 7-point self-measured blood glucose (SMBG) levels fail to accurately display the whole picture of blood glucose fluctuations that occur in a diabetic patient during the day. Professional continuous glucose monitoring (CGM) uses subcutaneous sensors and records the average interstitial sensor current every 5 min in the recorder; the data are subsequently exported to a computer 4 to 7 days later when calibration with SMBG is made retrospectively. The recorded results can display continuous fluctuations in blood glucose levels during the days when the patient carries the sensor. The patient must also record the blood glucose level 4 times a day for calibration of the blood glucose level. At the same time, the patient is requested to maintain a personal diet diary and record leisure activities so that the diabetologist can identify the reason for unexpected blood glucose fluctuation, which may result in suggestion of diet adjustment or antidiabetic drug adjustment. So far, professional CGM has been clinically used for patients with diabetes. Despite lack of strong evidence favoring professional CGM over SMBG in improving glycemic control, it is assumed that professional CGM is a tool for communication between physicians and patients to modify the treatment strategy [6].

In previous studies, real-time continuous glucose monitoring (RT-CGM) has been compared with SMBG and internet blood glucose monitoring (IBGM). There has been no significant difference between RT-CGM and IBGM in terms of their effects on HbA_1c_, and both have been shown to be better than SMBG alone [7,8]. However, although RT-CGM may provide better monitoring, subjects noted that wearing the CGM sensor is more likely to make them uncomfortable because it may cause conditions, including skin irritation and sleep disruptions, owing to the system alarm and thus may even cause subjects to become dissatisfied or prematurely end or refuse the use of RT-CGM [8]. In addition, previous studies on patients with type 1 diabetes have also suggested that other social factors may also be associated with the experience of using this monitoring system. First, the inconvenience of the monitoring system is a problem that must be solved. Patients who like the system find this problem to be tolerable. However, patients who do not like the system are heavily affected psychologically by its use and tend to have a poor user experience. Second, regarding the use of information, patients who are positive about the system suggest that this system may help increase their understanding and self-management of their glycemic status. However, patients who do not like the system think that too much information may not be relevant. Finally, care and encouragement from family and friends in the form of social support have a positive effect on the use of the CGM system as well as participant acceptance of the system. RT-CGM can also reduce the family’s anxiety about the disease, such as concern about hypoglycemia, and thus can improve the quality of life of patients [9]. In addition, when patients are more concerned about the control of blood glucose levels, they are not only willing to spend more time trying out this new technology but are also more willing to tolerate some of the discomfort caused by the system, such as skin allergy and irritations and alarm sounds [10].

### Objectives

Despite current research on the CGM system, most studies are conducted among patients with type 1 diabetes [9-12] using RT-CGM but not professional CGM [7-9,11]. In addition, current research on the CGM sensor has mostly focused on European countries and the United States [7-12]. At present, application of professional CGM among patients with type 2 diabetes in Taiwan is limited in the research field owing to its high cost that is not covered by the National Health Insurance (NHI) [13-15]. As a tool to aid in the management of type 1 diabetes [16], patients with type 1 diabetes can use the insulin pump along with RT-CGM to monitor their blood glucose levels, to reduce hypoglycemia, and to reduce insulin dosage [17] or to monitor preterm infants delivered by women affected by diabetes [18]. From the literature review, the perception of professional CGM among middle-aged and elderly patients with type 2 diabetes in non-Western countries is still lacking. Middle-aged and elderly people are very different from children or young adults in many aspects. They have different daily activities, social networks, and family support systems. Therefore, an exploration of the short-term and long-term acceptability of new technologies and whether they will bring about positive changes in behavior is urgently needed. Professional CGM (iPro 2) is the only available CGM device in Taiwan. We performed in-depth interviews to explore the acceptability and experience of professional CGM among middle-aged and older individuals and to explore the impact of professional CGM-based counseling on their health literacy and lifestyle.

## Methods

### Participant Selection

We recruited middle-aged and elderly patients with type 2 diabetes at an endocrinology outpatient department in a medical center in southern Taiwan. The inclusion criteria were as follows: patients with type 2 diabetes who were 45 years or older with inadequate controlled blood glucose (at least 2 of the last 3 HbA_1c_ readings at 7% or more) and patients who were suggested professional CGM as an interventional tool to improve their glycemic control by their primary care physician. Participants were excluded if they reported being diagnosed with generalized inflammation; advanced malignancy; end-stage renal disease on regular dialysis; status post renal transplantation; end-stage liver, heart, or pulmonary disease; or had any acute or chronic inflammatory disease as determined by a leukocyte count over 10,000/mm^3^ or clinical signs of infection. Patients diagnosed with thalassemia, glucose-6-phosphate dehydrogenase deficiency, or any other hemoglobinopathies that could influence the accuracy of the HbA_1c_ measurement were also excluded. In addition, patients who had HbA_1c_ levels above 12% at a recent outpatient visit were excluded owing to limitations of the CGM device to calibrate blood glucose above 400 mg/dL. Finally, participants who could not follow orders because of cognitive impairment or who were bedridden were also excluded. All participants provided written informed consent before the trial, and they also received compensation for their time. As for sample representation, the participants in our study were not limited to certain gender, occupation, educational level, duration of diabetes, or age. We tried to collect more information from participants in variable backgrounds. Besides, data collection continued until it was believed that data saturation had been achieved. The point of saturation was determined when new added data from participants no longer changed the researchers’ understanding about the topic.

### Continuous Glucose Monitoring Procedure

Participants wore professional CGM (iPro 2) for 5 days and measured their blood glucose at least 3 times a day for calibration of interstitial glucose readings. Participants were also requested to complete a diet diary with a photo record every day. After 5 days of wear, the sensor was removed. A semistructured in-depth interview for opinions about CGM and feelings during CGM was conducted on the day of the CGM sensor removal. The primary care physicians used CGM as a counseling tool to motivate patients to adjust their diet and exercise habit and to also make decisions on drug adjustment, if necessary, at the prescheduled outpatient visit. The second interview collected opinions about satisfaction of CGM and its influence on family members at the 3-month follow-up.

### Data Collection

A total of 2 semistructured in-depth interviews were conducted to collect information about the participants’ feelings and experiences related to CGM device usage. For the first interview, opinions about CGM and feelings when wearing the CGM device were explored on the day of the CGM sensor removal. For the second interview conducted at the 3-month follow-up when they visited the outpatient clinic, we focused on participant satisfaction with the device. All interviews were conducted by 2 trained researchers. We asked broad, open-ended questions about their opinions on the CGM device and adjusted the questions and asked for more details according to the flow of the conversation. Both the interviews were conducted for 15 to 20 min in a private room where participants received health education. We conducted the interviews at a familiar place to avoid effects due to unfamiliarity with the location of the interviews. All interviews were audio-recorded and transcribed verbatim. We analyzed the verbatim answers reported by the participants and categorized them into different concepts. The participants’ names and identifiers were removed to protect their confidentiality.

### Data Analysis

According to Dahlgren and Fallsberg’s recommendations [19], we listened to the interview content again to familiarize ourselves with the content and then transcribed it verbatim. We performed an analysis by labeling the content related to the structured discussion guide and comparing the content between different participants. Then, we categorized key words, phrases, and texts to determine the themes. We divided the participants’ answers into 3 topics: (1) participants’ adoption of the CGM device, (2) behavior while wearing the CGM device, and (3) can CGM be an effective behavioral modification tool? Finally, we concluded the core concept of each category and coded related quotes to explore the participants’ actual interaction with this new diabetes technology.

### Ethical Considerations

This study was approved by the Institutional Review Board of National Cheng Kung University Hospital on January 21, 2016 (IRB #B-ER-104-239).

## Results

### Overview

**Figure 1:**
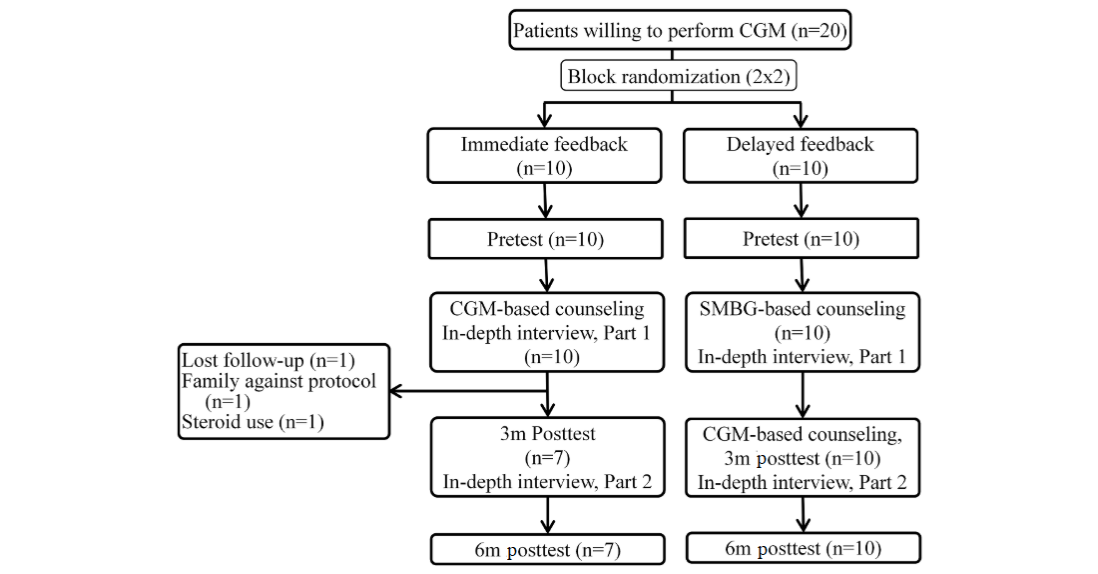
Enrollment flow diagram. CMG: continuous glucose monitoring; SMBG: self-measured blood glucose; 3m: 3 month; 6m: 6 month.

A total of 20 participants (13 males and 17 females) were recruited in this qualitative study. Figure 1 illustrates the details of the enrollment flow. Initially, all of the 20 participants signed informed consent forms and participated in the first part of the interview, which is about the opinions and feelings about CGM during 5 days of professional CGM exam. Among them, 17 participants completed the second part of the interview, which is about the satisfaction of CGM and influence of CGM on themselves and their family members.

As shown in Table 1, mean age of the 20 participants was 61 (SD 5) years, with the long-standing diabetes duration being 16 (SD 8) years, and body mass index 27.98 (SD 3.42) kg/m^2^. Before CGM, the mean HbA_1c_ among these participants was 8.54% (SD 0.71%), and the mean fasting blood glucose level was 177 (SD 48) mg/dl (Table 1).

There were 3 main areas that were explored in this study: (1) why they agreed to adopt the CGM device in their diabetes treatment, (2) their feelings related to incorporating technology into regular disease management behavior, and (3) by obtaining the attitude or behavioral changes before and after the CGM intervention, we tried to determine whether CGM is an effective behavior modification tool. Several themes for each question were identified (Table 2).

### Factors Related to Participants’ Adoption of the Continuous Glucose Monitoring Device

For this question, we explored the participants’ adoption of the device. We asked them about their motivation to participate in the trial and asked what factors affected their decision. In addition, we explored their initial perceptions of the device. According to their answers to each question, we categorized their responses into 2 themes as follows:

#### Theme 1: The Physician as an Authority Was Effective and Determinant

With regard to the participants’ adoption of the CGM device, professional authorities played an indispensable role. For the participants, the doctor assumed the role of a professional authority who was an information provider. Most of the participants had never heard of the device until doctors told them about it. The participants learned more about CGM from doctors or health education providers. At the same time, it increased participants’ motivation to receive the trial:

I participated because the doctor told me that it would be better to know what my blood glucose level was. My glycated hemoglobin was 7 [%] or so, but my [fasting] blood sugar level was 190 [mg/dl]. He said it was disproportionate, so I needed to wear this to know my blood glucose level.

No. 08, age 65, female

The doctor told me that it would help me understand the changes in my blood sugar level and how it functions... It could effectively monitor changes in my blood sugar level because in the past, we only measured blood sugar levels in the morning or after meals. So, to understand the real changes to my body condition, it would be more effective this way. My blood sugar levels were suddenly high and low, and I did not know whether the cause of the problem was something I ate or other bodily conditions. If I go through this, it should help me in some way. So, I gladly agreed.

No. 05, age 61, male

In addition, professional authorization was one of the important motivations that led participants to participate in the trial. Owing to their trust in a professional authority, participants were willing to give it a try:

I thought the doctor’s advice would be helpful. So, I said yes!...Owing to the physician’s enthusiasm, I felt that he was enthusiastic about helping me control my blood sugar levels and improve my body condition, and I did not want to lose the physician’s good will.

No. 06, age 56, female

However, professional authorities might have forced patients to participate in the trial. Of all the participants, 1 was afraid of rejecting the doctor’s recommendation because he felt that if he did not participate, the doctor would not provide him with medical care anymore.

Under these circumstances, he decided to participate in the trial:

There is not a specific reason. I am doing it because the physician suggested that I do this...We are not doctors, and how can we know? We just do whatever the doctor tells us to do.

No. 17, age 55, male

I dare not tell him I don’t want to! I cannot say it! I am afraid that the doctor will refuse my appointments in the future.

No. 03, age 62, male

#### Theme 2: Motivation to Solve Problems as a Key to Adoption

Besides professional authorization, most of the reasons for participation in the trial included the participants’ desire to understand their physical condition better. The participants looked forward to figuring out better treatment plans:

All I can answer is that I am doing this [experiment] for my own good! And I also want to know where the problem lies!

No. 08, age 65, female

### Feelings of Incorporating Technology Into Regular Disease Management Behavior

In this section, we explore the participants’ perceptions of CGM. We asked participants about their daily life while wearing the device and the role that it played. Did participants change their daily life, such as exercise and dietary habits, because of CGM? How did it affect their daily life? According to the participants’ answers to each question, we categorized them into 5 themes as follows:

#### Theme 1: Technology as a Reminder

In addition to participant perceptions, CGM also affected their behavior. For instance, it could serve as a reminder. Participants had to measure blood glucose 4 times every day. When they saw the blood glucose results, it reminded them about things such as changing their food intake, and the results also reminded them what they had eaten earlier in the day:

Well, if my blood sugar level is high, I will try to recall what I have eaten to make my blood sugar level so high, and then I would control my diet. For example, I would buy a baked scallion pancake that I really wanted to eat, but I would not eat it all at once. I would only take one bite or two, and I would wait for 1 or 2 hours to have another bite. It is like payment in installments. Ha!

No. 09, age 64, female

**Table 1:** Sample demographics (n=20).

Population demographic characteristic		Statistics	Range
Age (years), mean (SD)		61 (5)	53-72
Duration of diabetes (years), mean (SD)		16 (8)	4-40
**Gender, n (%)**			
	Male	13 (65)	—^a^
	Female	7 (35)	—
**Education, n (%)**			
	Elementary graduate	5 (25)	—
	High school graduate	10 (50)	—
	College education or greater	5 (25)	—
Body mass index (kg/m^2^), mean (SD)		27.98 (3.42)	22.40-34.37
Glycated hemoglobin (%), mean (SD)		8.54 (0.71)	7.3-10.0
Fasting blood glucose (mg/dl), mean (SD)		177 (48)	—
**Blood pressure (mm-Hg), mean (SD)**			
	Systolic pressure	138 (19)	110-203
	Diastolic pressure	84 (12)	60-115
**Physical activity, n (%)**			
	No	4 (20)	—
	1-2 times every week	5 (25)	—
	3-4 times every week	4 (20)	—
	>5 times every week	7 (35)	—
**Smoking, n (%)**			
	Yes	2 (10)	—
	No	18 (90)	—
**Drinking, n (%)**			
	No	17 (85)	—
	Occasionally	2 (10)	—
	Often	1 (5)	—

^a^Not applicable.

**Table 2:** Themes and answers to questions.

Themes	Participants’ adoption of CGM^a^	Perception of CGM	Can CGM be an effective behavior modification tool?
Theme 1	Physician’s advice was an effective determinant: “The doctor told me that it could help me understand the changes in my blood sugar level, and it functions in that way... It could effectively monitor changes in my blood sugar level; I asked many people, and they did not know much about this. We are not doctors, so how can we know this? We do whatever the doctor tells us to do”	Technology as a reminder: “Well, if my blood sugar level is high, I will try to recall what I have eaten to make my blood sugar level so high, and then I will control my diet.”	Visualization of blood glucose level helps with behavioral changes: “I have to adjust my diet. After a nap in the afternoon, I will have some chia seeds, and I do not eat white rice in the evening. I eat less in general, and the reduction in the amount of food intake really has a great association with my blood sugar levels; I used to measure it in the morning and evening, but now I measure it only once a day. I was worried that the blood sugar was too high, but now the blood sugar has stabilized, and I do not measure so many times. Otherwise, my hand will hurt from measuring my blood sugar level”
Theme 2	Individual problem-solving motivation is a key to adoption: “All I can answer is that I do this for my own good! And I also want to know where the problem lies!”	Technology as a supervisor: “Of course, I would be more cautious about my diet because I am wearing it.”; “One thing that I should pay attention to is that the device is still recording, so I have to be more careful about what I've eaten.”	Motivation at enrollment is a determinant: “It is OK as long as there is improvement to the control of my blood sugar levels! I would like to try it as long as it can help control my blood sugar levels.”; “No! Very few people have done this, and I asked many people and they did not know much about this. We are not doctors, so how can we know this? We do whatever the doctor tells us to do because it is too much of a bother to fill in the records.”
Theme 3	—^b^	Technology as a useful tool to visualize the blood sugar results: “Although I have insulin injections, I have no idea about my blood sugar levels. This time, after wearing the device, I know my own blood sugar levels”	—
Theme 4	—	Technology as an obstruction: “Because I had an operation on my waist before, I could easily get a backache. I used to have a hot bubble bath in the morning, but I cannot because I am wearing the device. It is causing some inconvenience, as my activity has become less smooth in the morning.”; “I feel it is very inconvenient! When I want to move things, I cannot use force. And I do not know how to use force because I am afraid I will break things.”	—
Theme 5	—	Technology as a hint of disease progression: “I just feel that medication is enough. Why do I need to go through this?”	—

^a^CGM: continuous glucose monitoring.

^b^Not applicable.

#### Theme 2: Technology as a Supervisor

Besides serving as a reminder, it also played the role of a supervisor. Some participants noted that they felt supervised when they used the CGM. They realized that they had to follow the principles of a diabetes diet because the CGM could record everything they had done. Therefore, they ate less or did not have snacks during the trial. They followed the diabetes diet when wearing the CGM device:

One thing that I paid attention to was that the device was still recording, so I had to be more careful about what I had eaten, and I think it's good because when I did not wear it [CGM], I did not know what I ate and what was happening to my body, and then when I wore it, it helped me record my blood sugar levels, and I knew I would like to eat less of something. I tried my best to eat more and eat better.

No. 06, age 56, female

It [diet] is normal, and I may be more restrained. I will control myself more when it comes to eating fruits or drinking beverages.

No. 10, age 67, male

#### Theme 3: Technology as a Useful Tool to Visualize the Blood Sugar Results

The CGM device provided a visualized outcome of the participants’ blood glucose levels. Most participants mentioned that they realized the relationship between their food intake and changes in their blood glucose levels. Some participants said that they had received health education, so they now realized what they should eat. However, the relationship between food intake and the changes in blood sugar was not clear until they participated in the trial:

This time, after wearing the device, I know my own blood sugar levels...In the past, I only measured my blood sugar levels once a week in the morning before breakfast. But this time, I know that blood sugar levels differ in the morning before a meal and before the time to go to bed...I did not know about this before...Because this device measures the blood sugar level every day in the morning and at night, I know that it is normal for my blood sugar level to be higher at 140 or 150 [mg/dl] in the morning. Otherwise, I used to wonder why it [blood glucose level] was so high despite my efforts to control my blood sugar level.

No. 12, age 67, female

#### Theme 4: Technology as an Obstruction

Though it acted as a reminder, a supervisor, and a visualizer, the CGM device also created some problems for some participants. To complete the trial, participants had to record everything that they ate; some participants could not complete it by themselves. They had to ask their family members for help:

Usually it is okay, but sometimes it is necessary for me to write the records. I don’t have a high level of education, so sometimes, I have to ask my husband to help me write the records, so it’s troublesome.

No. 08, age 65, female

In addition, 1 participant noted that he could only take a shower instead of taking a bath when wearing the CGM device. Owing to this, he could not relieve his back pain:

Because I had an operation on my waist before, I can easily get a backache. I used to have a hot bubble bath in the morning, but I cannot because I am wearing the device. It is inconvenient now that my activities have become less smooth in the morning.

No. 05, age 61, male

Moreover, the CGM device could also be an obstruction for participants. When they wore the device, their body movements were affected:

I feel it is very inconvenient! When I want to move things, I cannot use force, and I do not know how to use force because I am afraid of breaking things.

No. 13, age 62, male

#### Theme 5: Technology as a Hint of Disease Progression

For the participants, the CGM device was not only a medical intervention to improve health management but it was also a hint of disease progression. This medical intervention revealed when the participants’ physical condition became so worse that the CGM device had to be used:

I am in a bad mood because I just feel that medication is enough. Why do I need to go through this?

No. 03, age 62, male

### Can Continuous Glucose Monitoring Be an Effective Behavioral Modification Tool?

To answer this question, we explored the participants’ behavioral changes before and after wearing the CGM device. We asked them whether their perceptions of CGM changed or if their disease management became different. We categorized the participants’ answers into 2 themes as discussed below:

#### Theme 1: Visualization of Blood Glucose Level Supports Behavioral Change

Technology brought confidence and self-efficacy that helped the participants to measure their blood glucose levels more effectively. The participants had typically only measured their blood sugar in the morning in the past. However, some participants mentioned that they would like to measure it at different times in a day to observe the changes in their blood sugar levels over time:

I used to take a measurement in the morning, so I did not know that the blood glucose levels could be different at different time points...After wearing this, I want to measure my blood sugar levels before breakfast, lunch, and dinner.

No. 12, age 67, female

However, the opinions may have been very different because when the participants realized that glycemic control was stable through this technologic intervention and they had more self-confidence in glycemic control, they decreased the frequency of measuring their blood glucose:

I used to take a measurement in the morning and evening, but now I measure it only once a day. I was worried that the blood sugar was too high, but now the blood sugar has stabilized, and I do not measure it so many times. Otherwise, my hand will hurt from measuring the blood sugar level.

No. 21, age 57, female

It also helped visualize the blood glucose levels so that participants knew how to modify their dietary behavior. Participants changed the kinds of food that they ate or reduced the amount of food that they ate. In addition, they paid attention to their diet:

I have to adjust my diet. After a nap in the afternoon, I will have some chia seeds, and I do not eat white rice in the evening. I eat less in general. The reduction in the amount of food intake really has a great association with the blood sugar levels...Now, I also need to reduce my juice intake!

No. 18, age 69, male

I will start to change my life habits slowly! Because I have a table...My doctor just gave it to me too. From this, I can see when my blood sugar is relatively high or low and what I can or cannot eat. I will adjust my diet based on it.

No. 12, age 67, female

#### Theme 2: Motivation at Enrollment Is a Determinant

Participants’ behavior while wearing the CGM device was related to their motivation to participate in the trial. One of the motivations was that they wanted to understand their physical condition and know how to improve glycemic control. The other was that they trust their doctors or they are embarrassed to reject a doctor’s suggestion.

The more desire that the participants had, the more actively they participated in the trial. Some participants mentioned that they were willing to do anything that is good for their health, and the CGM device was no exception:

I would like to try it as long as it can help control my blood sugar levels...I will pay attention to my diet. I would like to try if it helps control my blood sugar levels when I make adjustments to my diet. From this test, I really find that there is quite an influence...It [adjustment of diet] has improved control of my blood sugar levels a lot. Look at my blood sugar levels. I have never had a level less than 100 [mg/dl].

No. 01, age 61, male

In addition to following the original trial procedure, participants may do something different from their normal food intake to achieve more changes in their blood sugar. This trial was like another experiment. In addition to their regular routines, participants may do something different to find a better way to control their blood sugar:

I mainly want to know how my blood sugar level can rise so much...I should go through a test to see what I should not eat...I even heard that there was a kind of herb that is good for the treatment of diabetes. I am planting this kind of herb. I thought, why not give it a try? Yesterday, I ate some pieces of the herb. This morning, I also ate some pieces, and my blood sugar level is indeed reduced.

No. 18, age 69, male

The participants’ opinions on the CGM device were also associated with their motivation to participate in the trial. The participants who actively wanted to be involved in the trial were more cooperative during the trial. Despite many trivial details, they were willing to complete the trial. On the contrary, participants who passively participated in the trial were inclined to complain about trivial details and be in a bad mood:

Quite honestly, I think I am also a co-operative patient...but the control of my blood sugar levels is not very satisfactory...So, I want to find out the problem through this test and avoid it in the future to see if it can really improve the condition of my body...It (measuring the blood glucose level several times a day) is not so bothersome because I used to measure my blood sugar level each day. Although this device measures my blood sugar level at a slightly higher frequency, I do not need to pay attention to the time for measurement of my blood sugar level, so it does not affect me that much.

No. 05, age 61, male

I am in a bad mood because I just feel that medication is enough. Why do I need to go through this...I do not like the feeling of being controlled. My doctor told me that if I wanted to control my blood sugar level well, I had to go through blood glucose monitoring...And I did not dare to refuse him...I cannot go out for lunch because I need to monitor the blood sugar level after eating...I am afraid that I will lose my freedom!

No. 03, age 62, male

Very few people have done this (CGM), and I asked many people, and they did not know much about this. We just do whatever the doctor tells us to do...It [the experimental procedure] is too much of a bother to fill in the records. It's not easy to record what you have eaten if it was just a snack. For example, do I have to make a record even when I only have two or three peanuts? I think it is hard to record everything! This is too troublesome!

No. 19, age 69, male

## Discussion

### Principal Findings

This is the first study in a non-Western country exploring the impact of a professional CGM system on middle-aged and older patients with type 2 diabetes. Patients aged 45 years or older who received CGM-based counseling were interviewed. Older patients’ perceptions related to incorporating technology into their diabetes care, attitude and behavioral changes related to the technology, immediately and 3 months after CGM usage, were explored. Gender differences were also found in this study.

Our findings suggest that the physician is the dominant and most effective information provider, and most of the participants gained access to this new technology via their physician. In addition, patients’ trust in their physicians made them want to try this new technology because they thought that their doctor would choose the best management for them. In a past study, there was also some mention of this opinion. A qualitative study exploring medication use in seniors indicated that a doctor is a trusted authority. On the basis of this trust, people felt confident that their doctor was choosing medications best suited for them [20]. The attitude of health providers is a dominant factor in patient disease management as well [21]. In addition, health care personnel play a vital role in adoption of new technology. Doctors may be information providers, and they also increase access for patients to new technology.

However, physician attitude or insufficient training also can be a barrier to new technology use. Some endocrinologists view CGM as a waste of money or have little information about it. These negative perceptions also affect adoption of CGM in diabetes patients [22,23]. In our study, we explored another adverse effect that results from professional advice. One of our male participants reported that CGM could be a hint of disease progression. Therefore, his perceptions of this device were negative. We assess that this could be a negative effect derived from professional opinions. The misconception of disease progression may result from doctors’ unclear explanations or information. Thus, these findings suggest a potential connection between professional advice and middle-aged and older patients’ adoption of new technology. When it comes to application of new technology, professional authorities may play an important role.

In chronic illness management, social support plays a vital role. Sufficient social support benefits disease self-management [24]. In our study, the participants revealed that CGM could be a reminder or supervisor that helped them to follow their original diet plan. The device was like another person reminding them and helping them have better diet control. In a past study, it was also reported that insufficient social support or overbearing support is also a source of patient distress [25]. In addition to true interactions between people, technology is gradually influencing disease management. It can be a reminder to help patients adhere to their plan [26]. For these reasons, we suggest that technology may be an effective part of chronic illness management.

In our study, we also found a gender difference in the attitude toward technology as indicated in previous research [27]. There was a higher percentage of women compared with men who reported that CGM played a role as a reminder or a supervisor to help them with dietary control. Among all participants, 4 of 7 (57%) women had this perception, whereas only 4 of 13 (30%) men had the same opinion. A previous CGM study for women showed the same result. The majority of women reported they were interested in changing their diabetes-related self-care behavior [28]. In a past study, it was revealed that social support is also gender-related. For men, coping with diabetes is strongly affected by their living spouse, and men receive more support from their spouse for dietary needs than women receive from their spouse [24,29]. However, females actually exhibit a greater psychological impact of diabetes than males [29]. In addition, females may gain more benefits from social support than men [30]. Under these circumstances, we suggest that women may need more social support to have better self-management. Therefore, we suggest that technology intervention may make up for women’s lack of social support.

### Limitations

The limitations of this study include the fact that it was a small, homogenous sample. All participants were from a city in southern Taiwan. For this reason, access to health education or disease information was almost the same for the entire sample. In addition, the participants were only partially selected. Our study had to be reviewed by the Institutional Review Board and Ethics Committee. Therefore, the participants were all informed before they were recruited. These participants might have been more motivated to use new technology. Thus, there might have been a bias. Moreover, the interviews were conducted individually by 2 people, and every interview did not take an equal amount of time, which might have led to some bias.

### Conclusions

In conclusion, this study identified perceptions and usage experience of professional CGM in middle-aged and older patients with type 2 diabetes. The participants’ problem-solving motivation and the advice of professionals were determinants of adoption of a new technology. Professional CGM helps visualize glucose control generally. We also found that technology intervention could be an effective behavioral modification tool and support system with the 3-month follow-up interviews. In addition, there was a higher percentage of women compared with men who reported that CGM played a role as a reminder or a supervisor to positively help them with dietary control. As type 2 diabetes is a highly behavioral modification and support-needed disease, and the fact that social support is gender-related, our findings that a technology intervention can make up for lack of social support, especially for women, warrant future verification.

## Abbreviations

CGM: continuous glucose monitoring
HbA_1c_: glycated hemoglobin
IBGM: internet blood glucose monitoring
RT-CGM: real-time continuous glucose monitoring
SMBG: self-measured blood glucose
